# Influence of vitamin D binding protein isoforms on the total and free serum 25-hydroxyvitamin D responses to vitamin D supplementation in older adults in the Multi-Ethnic Study of Atherosclerosis

**DOI:** 10.3389/fendo.2026.1910020

**Published:** 2026-10-29

**Authors:** Xinran Hu, Andrew N. Hoofnagle, Simon Hsu, David K. Prince, Mark M. Kushnir, Alexis C. Wood, Stephanie Sisley, Erin D. Michos, Bruce M. Psaty, Steven J. Shea, Jerome I. Rotter, David Siscovick, Norrina B. Allen, Russell P. Tracy, Karol E. Watson, Xiaohui Li, Kent D. Taylor, Bryan R. Kestenbaum, Ian H. de Boer, Cora M. Best

**Affiliations:** 1 Food Science and Human Nutrition Department, Institute of Food and Agricultural Sciences, University of Florida, Gainesville, FL, United States; 2 Kidney Research Institute, Department of Medicine, University of Washington, Seattle, WA, United States; 3 ARUP Institute for Clinical and Experimental Pathology, University of Utah, Salt Lake City, UT, United States; 4 Department of Pediatrics, Children's Nutrition Research Center USDA/ARS, Baylor College of Medicine, Houston, TX, United States; 5 Division of Cardiology, Department of Medicine, Johns Hopkins University, Baltimore, MD, United States; 6 Cardiovascular Health Research Unit, Departments of Medicine, Epidemiology and Health Systems and Population Health, University of Washington, Seattle, WA, United States; 7 Department of Medicine, Columbia University Vagelos College of Physicians and Surgeons, New York, NY, United States; 8 The Lundquist Institute for Biomedical Innovation at Harbor-UCLA Medical Center, Torrance, CA, United States; 9 New York Academy of Medicine, New York, NY, United States; 10 Department of Preventive Medicine, Northwestern University, Chicago, IL, United States; 11 Department of Biochemistry, University of Vermont, Burlington, VT, United States; 12 Department of Medicine, David Geffen School of Medicine at UCLA, Los Angeles, CA, United States

**Keywords:** 25-hydroxy vitamin D, free 25-hydroxy vitamin D, vitamin D binding protein (GC), vitamin D binding protein gene, vitamin D deficiency, vitamin D_3_

## Abstract

**Introduction:**

Genetic variants that determine the vitamin D binding protein isoform (GC) are consistently associated with total serum 25(OH)D concentration in cross-sectional genome-wide association studies. Previous studies suggest GC isoforms may influence the increment of total 25(OH)D in response to vitamin D supplementation. The free serum 25(OH)D concentration may better reflect vitamin D status, but whether GC isoforms modify the effect of supplementation on free 25(OH)D requires further study.

**Objectives:**

This study aimed to determine the associations between GC isoforms and the increments of total and free 25(OH)D_3_ in response to vitamin D_3_ supplementation among older adults.

**Methods:**

Data were derived from the Multi-Ethnic Study of Atherosclerosis Individualized Response to Vitamin D Treatment (MESA INVITe) Study (NCT02925195). Participants (n = 666) were randomized 3:1 to 16 weeks of 2,000 IU of vitamin D_3_ or placebo daily. Complete data from 559 participants (414 vitamin D_3_; 145 placebo) were used for analysis. Total serum 25(OH)D_3_ concentration, GC isoforms, and serum VDBP concentration were determined by LC-MS/MS. Free serum 25(OH)D_3_ was measured by LC-MS/MS following sample preparation by size-exclusion separation. Additionally, an established prediction equation was used to estimate free 25(OH)D_3_ from common biochemical measurements. The associations of GC isoforms with baseline, endpoint, and 16-week changes in total and free 25(OH)D_3_ were modeled with linear regression. Models were adjusted for sex, age, season, body mass index, estimated glomerular filtration rate, and genetic ancestry.

**Results:**

Mean age (SD) of the participants was 72 (8) y; 53% were females, 36% White, 33% Black, 18% Hispanic/Latino, and 13% Chinese. In each treatment group at both baseline and endpoint, total 25(OH)D_3_ was lower among individuals carrying GC2 isoform (3 to 6 ng/mL lower per GC2 allele), whereas free 25(OH)D_3_, whether measured or calculated, did not vary according to GC isoform. Supplementation significantly increased total and free 25(OH)D_3_, but the increments of total and free 25(OH)D_3_ were independent of GC isoform.

**Discussion:**

At baseline and endpoint, total serum 25(OH)D_3_ concentration, but not free 25(OH)D_3_, was lower among individuals with GC2 isoform. However, neither the total nor free 25(OH)D_3_ response to vitamin D_3_ supplementation varied according to GC isoform.

## 1 Introduction

The prohormone 25-hydroxyvitamin D (25[OH]D) is the most abundant circulating vitamin D metabolite in humans, and the concentration of total (protein-bound plus unbound) 25(OH)D in serum/plasma is used to assess vitamin D status in clinical settings. However, it is hypothesized that the unbound (i.e., free) 25(OH)D concentration may be a more accurate biomarker of vitamin D status that is not influenced by alterations in plasma protein binding.

The majority of vitamin D and its hydroxylated metabolites circulate bound to vitamin D binding protein (VDBP) (~85-90%) and albumin (~10-15%) (1). Only 0.02-0.04% of 25(OH)D and 0.3-0.4% of 1,25-dihydroxyvitamin D (1,25[OH]_2_D) are unbound in the circulation (2). The “Free Hormone Hypothesis” states that only vitamin D molecules that are not bound to VDBP or albumin can enter most target cells by diffusing through the plasma membrane (2). Results from experiments with VDBP-null mice and clinical data from rare cases of homozygous deletion of the *GC* gene that encodes VDBP lend support to a free vitamin D hypothesis. VDBP knockout mice had severely low circulating total 25(OH)D. However, when VDBP KO mice consumed a vitamin D-replete diet, there were no signs of vitamin D deficiency, and they showed normal tissue levels of 1,25(OH)_2_D and expression of vitamin D-responsive genes, suggesting that free 25(OH)D sustained biological activity (3, 4).

The first human case of *GC* gene deletion was reported in 2019 in a 58-year-old woman with severe ankylosing spondylitis and complete VDBP deficiency (5). Despite nearly undetectable vitamin D metabolite concentrations that did not respond to supplementation, she maintained normal calcium levels and only mild bone disturbances, indicating VDBP is not essential for calcium balance. This and other (6, 7) rare clinical cases support the free hormone hypothesis for vitamin D, including that total vitamin D measurement can be misleading in VDBP-deficient individuals, and suggest that free 25(OH)D is the critical determinant of vitamin D biological activity and sufficiency.

Prior evidence relating free 25(OH)D to functional and clinical outcomes is mixed, and better methods to determine free 25(OH)D are needed (8). Measuring free (unbound) vitamin D metabolites is challenging primarily because less than 1% of these metabolites circulate in the free form. Commonly used assays like ELISA can suffer from issues like cross-reactivity and interference from protein-bound forms. There is also a lack of standardized methods and reference ranges for free 25(OH)D, and there remains ongoing debate about its clinical relevance. All these factors contribute to the complexity and limited routine use of free vitamin D measurement in clinical practice. Some mathematical models have been developed to predict or calculate free 25(OH)D based on other biochemical measurements, typically total 25(OH)D, VDBP, and albumin concentrations and published affinity constants of 25(OH)D_3_ for the binding proteins (2, 9). However, the affinity of 25(OH)D for VDBP may differ according to GC isoform, and other factors that influence the ligand-protein binding such as life stage, clinical condition, and medication are unaccounted for in the published equations (10).

The GC isoforms of the vitamin D binding protein are genetic variants that influence vitamin D concentration and, potentially, bioavailability. These isoforms arise from polymorphisms in the *GC* gene, primarily the SNPs rs7041 and rs4588, which are associated with ancestry and result in three haplotypes (GC1f, GC1s, and GC2) inherited to yield 6 diplotypes (**Supplementary Table 1**) (11). GC isoforms are thought to modify the binding of 25(OH)D in circulation. In cross-sectional genome-wide association studies, the polymorphisms that determine these GC haplotypes have some of the largest single variant effects on total serum 25(OH)D and VDBP concentrations, with GC2 consistently associated with lower concentrations (12–15). Furthermore, binding affinity may differ according to the isoform (16).

From first principles, both VDBP concentration and binding affinity influence the free fraction of circulating 25(OH)D. Because 25(OH)D is highly protein-bound and slowly metabolized, differences in the free fraction are likely to translate to differences in systemic clearance of 25(OH)D (17) and, potentially, the increase in circulating total 25(OH)D concentration in response to oral vitamin D supplementation. However, the influence of GC isoforms on the 25(OH)D response to supplementation remains unclear. Several studies have shown that individuals with the rs4588 minor allele (GC2 haplotype) had a lower response to vitamin D supplementation, but this finding was not replicated in all studies (18, 19). In contrast, the variant rs7041 (GC1s) has been associated with either a greater or comparable increase of 25(OH)D in response to the vitamin D supplementation, depending on the rs7041 variant present (20, 21). Inconsistencies may result from variations in study population, supplementation duration, supplementation dose, and statistical modeling. Nevertheless, because most prior supplementation studies measured only total 25(OH)D, it remains unclear whether GC isoforms affect the measured free 25(OH)D response, which is important for evaluating genotype-guided vitamin D dosing.

The primary aim of this study was to evaluate changes in total and free 25(OH)D_3_ with supplementation according to GC isoforms. We hypothesized that the total 25(OH)D_3_ response would vary according to GC isoforms due to differences in the protein binding, but the free 25(OH)D_3_ response would not. This study provides insight into the potential for precision vitamin D dosing based on an individual's GC isoform (genotype).

## 2 Materials and methods

### 2.1 Study design

The INdividual response to VITamin D (INVITe) trial was a randomized, double-blind, placebo-controlled trial that was conducted during 2017–2019 and was nested in an ongoing nationwide cohort study, the Multi-Ethnic Study of Atherosclerosis (MESA). The INVITe trial was designed to evaluate whether clinical and genetic factors play a role in modifying the effects of vitamin D_3_ supplementation (22). The study protocol and evaluations of the clinical factors and common genetic variants associated with the biochemical response to oral vitamin D_3_ have been published (22–24).

### 2.2 Study population

From 2000 to 2002, MESA recruited participants from six United States communities and asked participants to self-report as White, Black, Hispanic/Latino, or Chinese. During the 2000–2002 MESA recruitment, there were 6,814 adults aged between 45–84 years old who enrolled in MESA. For the INVITe trial, participants were recruited from the 15^th^ year MESA examination (Exam 6) or from two parallel MESA ancillary studies (MESA Family and MESA Air). Participants were recruited from five field centers (Los Angeles County, CA; Chicago, IL; Baltimore and Baltimore County, MD; Forsyth County, NC; and Northern Manhattan and the Bronx, NY). Exclusion criteria included use of high-dose vitamin D supplements (>1,000 IU/day), calcitriol, or calcitriol analogues; history of a clinical diagnosis of primary hyperparathyroidism or sarcoidosis; kidney stones within the previous 5 years; history of kidney transplantation or kidney replacement therapy; serum calcium concentration >11 mg/dL at the MESA examination conducted between 2000 and 2002; or self-reported history of elevated serum calcium concentration. The study was approved by the Institutional Review Board at each site and was registered prior to initiation (NCT02925195). All participants provided written informed consent.

### 2.3 Intervention

Participants were randomly assigned to the vitamin D_3_ supplement or placebo groups at a ratio of 3 (vitamin D_3_) to 1 (placebo). Participants received either 2,000 IU of vitamin D_3_ or a masked placebo daily for 16 weeks.

### 2.4 Outcomes

The current analysis evaluated total and free serum 25(OH)D_3_ concentrations in the participants at baseline and at 16 weeks, and the changes in these analyte concentrations. Our primary aim was to determine whether these outcomes varied according to GC1s or GC2 isoform carriage. Total (bound plus unbound) serum 25(OH)D_3_ concentration was measured using LC-MS/MS; the total mean CV% for total 25(OH)D was 4.3% (23). Further assay performance metrics have been published previously (25).

Concentrations of free 25(OH)D_3_ were measured using a novel mass-spectrometry–based method that employs size-exclusion separation to isolate free from protein-bound 25(OH)D_3_, followed by derivatization and quantification by LC-MS/MS (17, 26). The lower limit of quantitation for the LC-MS/MS method for free 25(OH)D_3_ was 5 pg/mL; total mean CV% was <18% at concentrations 5–50 pg/mL and <10% at concentrations >50 pg/mL (27, 28). Additionally, a published prediction equation that has been applied widely in prior research (referred to hereafter as Bikle’s equation) was used to estimate free 25(OH)D_3_ (29):


$$free\;vitamin\;D\;metabolite=\frac{Total\;vitamin\;D\;metabolite}{1+[Ka_{alb}*albumin]+[Ka_{DBP}*DBP]}$$

Serum albumin was measured with a Beckman-Coulter AU 5812 analyzer. The serum VDBP concentration and GC isoforms were determined directly by LC-MS/MS (23, 30); the total mean CV% for determining VDBP concentration was 3.6% (23). For quality assurance, the isoform data were compared with genotypes imputed from microarray data obtained for the parent MESA study (31).

It has been proposed that the molar ratio of 24,25(OH)_2_D-to-25(OH)D (vitamin D metabolite ratio, VDMR) is a functional measure of vitamin D bioactivity that is independent of vitamin D binding protein concentration (32). We hypothesize that its assessment may closely mirror that provided by the measurement of free 25(OH)D_3_. Thus, VDMR was evaluated in this study as an exploratory outcome. Serum 24,25(OH)_2_D_3_ was measured simultaneously with 25(OH)D_3_ using a multiplexed LC-MS/MS assay. The total mean CV% for 24,25(OH)_2_D_3_ was 7.5% (23, 25, 33).

### 2.5 Covariates

Model covariates included age, sex, season, BMI, eGFR, and the top 5 principal components of genetic ancestry. At the start of the MESA study, participant information such as race and ethnicity, age, and sex was gathered through self-completed surveys (34). The top 5 principal components of genetic ancestry were determined using SMARTPCA from EIGENSOFT (24). The first five principal components were used in the models to strike a balance between conservative control for population stratification and model parsimony. Height and weight were recorded to compute body mass index (BMI) in kilograms per square meter. Kidney function, as estimated glomerular filtration rate (eGFR), was previously calculated from serum creatinine using the CKD-EPI 2009 formula (35). The season of the INVITe baseline exam was categorized into 4 bins: January-March, April-June, July-September, and October-December (36).

### 2.6 Statistical analysis

The Agreement Test in R (version 4.3.1) was used to compare the GC isoform results between LC-MS/MS and microarray. The free 25(OH)D_3_ methods (directly measured by LC-MS/MS and estimated by Bikle’s prediction equation) were compared by using Deming regression and Bland-Altman analyses.

In each treatment group separately, we used multivariable linear regression to examine the associations of baseline, 16-week, and 16-week change in total and free 25(OH)D_3_ with GC2 and GC1s isoform carriage. To take further advantage of the presence of a placebo group, we also tested whether the effects of supplementation on 16-week changes in total and free 25(OH)D_3_ differed according to GC1s or GC2 isoform carriage using an interaction term between the GC isoform and treatment assignment (*i.e.*, tested for effect modification).

## 3 Results

### 3.1 Participants characteristics

In the INVITe trial, 666 participants were enrolled, and 559 with complete data were analyzed here. 55 participants were excluded from this analysis due to missing outcome data (they did not complete the study). Another 49 were excluded from this analysis due to missing covariate data, and 3 were removed because the GC isoform results contradicted the imputed genotype (**Supplementary Figure 1**). Among the 559 participants in this analysis, 549 had completed pill counts; the median (IQR) adherence to study medications was 98% (91, 100). The average age within the analytic sample was 72 years. Among all the participants, 36% were White, 33% were Black, 18% were Hispanic/Latino, and 13% were Chinese (**Table 1**). Baseline characteristics were similar between the vitamin D_3_ treatment group and placebo group (**Table 1**). At baseline, the mean concentration of serum 25(OH)D_3_ in the vitamin D_3_ treatment group was 28.9 ± 11.6 ng/mL, serum 1,25(OH)_2_D_3_ was 48.7 ± 18.7 pg/mL, and serum PTH was 49.1 ± 29.6 pg/mL. Among the placebo group, the mean baseline serum 25(OH)D_3_ was 28.2 ± 10.5 ng/ml, serum 1,25(OH)_2_D_3_ was 48.2 ± 16.4 pg/mL, and serum PTH was 43.4 ± 20.7 pg/mL. The prevalences of GC1s, GC1f, and GC2 isoform carriage were 345 (61.7%), 317 (56.7%), and 204 (36.5%), respectively. The distribution of GC diplotype groups is shown in **Table 1**.

**Table 1:** Baseline characteristics of participants in the analysis^1^.

	D3 N= 414	Placebo N= 145	All N= 559
Age, mean (SD), years	72.3 (8)	71.4 (8)	72.1 (8)
Female, n (%)	225 (54.3)	71 (49.0)	296 (53.0)
Race and ethnicity, n (%)			
White	148 (35.7)	53 (36.6)	201 (36.0)
Black	135 (32.6)	47 (32.4)	182 (32.6)
Hispanic/Latino	73 (17.6)	27 (18.6)	100 (17.9)
Chinese	58 (14.0)	18 (12.4)	76 (13.6)
Study site, n (%)			
Forsyth County, NC	32 (7.7)	14 (9.7)	46 (8.2)
Northern Manhattan and the Bronx, NY	121 (29.2)	37 (25.5)	158 (28.3)
Baltimore and Baltimore County, MD	85 (20.5)	30 (20.7)	115 (20.6)
Chicago, IL	108 (26.1)	40 (27.6)	148 (26.5)
Los Angeles, CA	68 (16.4)	24 (16.6)	92(16.5)
Annual gross family income, n (%), $			
<20,000	64 (15.5)	13 (9.0)	77 (13.8)
20,000 to <35,000	78 (18.8)	34 (23.4)	112 (20.0)
35,000 to <75,000	132 (31.9)	41 (28.3)	173 (30.9)
≥75,000	140 (33.8)	57 (39.3)	197 (35.2)
Season at MESA INVITe baseline exam, n (%)			
January - March	65 (15.7)	23 (15.9)	88 (15.7)
April - June	99 (23.9)	35 (24.1)	134 (24.0)
July - September	135 (32.6)	47 (32.4)	182 (32.6)
October - December	115 (27.8)	40 (27.6)	155 (27.7)
Non-study vitamin D supplements^2^, n (%)			
None	240 (58.0)	95 (65.5)	335 (59.9)
1–400 IU daily	23 (5.6)	11 (7.6)	34 (6.1)
401–1000 IU daily	148 (35.7)	37 (25.5)	185 (33.1)
NA	3 (0.7)	2 (1.4)	5 (0.9)
Ever smoker, n (%)	209 (50.5)	80 (55.2)	289 (51.7)
BMI, mean (SD), kg/m2	28.8 (5.7)	28.4 (5.3)	28.7 (5.6)
Diabetes, n (%)	85 (20.5)	27 (18.6)	112 (20.0)
Hypertension, n (%)	273 (65.9)	80 (55.2)	353 (63.1)
Prevalent cardiovascular disease, n (%)	23 (5.6)	5 (3.4)	28 (5.0)
eGFR, mean (SD), mL/min/1.73m2	77.1 (17.1)	80.3 (14.3)	77.9 (16.5)
Serum Total 25(OH)D_3_, mean (SD), ng/mL	28.9 (11.6)	28.2 (10.5)	28.7 (11.3)
Serum 25(OH)D_2_, median(Q1-Q3), ng/mL	0.4 (0.3-0.8)	0.4 (0.3-0.8)	0.4 (0.3-0.8)
Serum 1,25(OH)_2_D_3_, mean (SD), pg/mL	48.7 (18.7)	48.2 (16.4)	48.5 (18.2)
Serum 24,25(OH)_2_D_3_, mean (SD), ng/mL	1.9 (1.1)	1.8 (1.0)	1.9 (1.1)
VDMR	0.06 (0.02)	0.06 (0.02)	0.06 (0.02)
Serum PTH, mean (SD), pg/mL	49.1 (29.6)	43.3 (20. 7)	47.6 (27.7)
Serum albumin, mean (SD), g/dL	4.1 (0.2)	4.1 (0.3)	4.1 (0.3)
Serum calcium, mean (SD), mg/dL	9.4 (0.4)	9.4 (0.4)	9.4 (0.4)
VDBP isoforms, n (%)			
GC1s/GC1s	89 (21.5)	23 (15.9)	112 (20.0)
GC1s/GC2	74 (17.9)	33 (22.8)	107 (19.1)
GC1s/GC1f	87 (21.0)	39 (26.9)	126 (22.5)
GC2/GC2	18 (4.3)	5 (3.4)	23 (4.1)
GC2/GC1f	56 (13.5)	18 (12.4)	74 (13.2)
GC1f/GC1f	90 (21.7)	27 (18.6)	117 (20.9)

^1^ BMI, body mass index; eGFR, estimated glomerular filtration rate; VDMR, vitamin D metabolite ratio; PTH, parathyroid hormone; VDBP, vitamin D binding protein.

^2^ Mean daily intake from all reported supplements.

### 3.2 Methods comparison

The agreement between LC-MS/MS and microarray for rs7041 genotype, which confers GC1s isoform, was 98.54%; for rs4588, which confers GC2, the agreement was 98.89%. Results from the LC-MS/MS method and Bikle’s prediction method used to determine free 25(OH)D_3_ were positively correlated, but the two methods did not agree well. Deming regression of the directly measured free 25(OH)D_3_ concentration regressed on the calculated concentration produced the equation Y = 11.3 x – 62.9 (**Supplementary Figure 2**). The Pearson correlation coefficient comparing those variables was 0.62 (p-value is < 2.2E-16), which indicates a moderate-to-strong positive linear relationship. The mean difference between the directly measured and calculated free 25(OH)D_3_ was 34 pg/mL, which indicates that the directly measured free 25(OH)D_3_ level tends to be higher than the Bikle’s calculated level. (**Supplementary Figures 2**, **3**).

### 3.3 Total 25(OH)D_3_

Among all participants at baseline, GC2 isoform carriage was associated with lower total 25(OH)D_3_ concentration. The model-based mean (standard error) total 25(OH)D_3_ was 30.0 (0.6) ng/mL, 26.6 (1.2) ng/mL, or 24.4 (2.6) ng/mL among participants with zero, one, or two GC2 alleles, respectively (p = 0.004 from Wald test for trend across genotypes). Within each treatment group, GC2 isoform carriage was still significantly associated with lower baseline total 25(OH)D_3_ concentration, although only when comparing GC2 heterozygotes to non-carriers, possibly due to the limited number of GC2 homozygotes (**Table 2**). After 16 weeks of vitamin D_3_ supplementation, the total 25(OH)D_3_ concentration remained significantly lower in individuals carrying the GC2 isoform (**Table 2**). By contrast, GC1s carriage was not associated with either baseline or post-supplementation total 25(OH)D_3_ concentration (**Table 3**). In the vitamin D_3_ group, total 25(OH)D_3_ increased by a mean (SD) 10.99 (9.4) ng/mL (**Figure 1A**). Contrary to our hypothesis, the magnitude of the increase did not vary according to GC isoform carriage (**Tables 2**, **3**).

**Table 2:** Changes in total and free serum 25(OH)D3 in response to vitamin D3 supplementation according to GC2 isoform carriage^a^.

		N		Baseline				16 week				Change after 16 weeks				Treatment effect modification by GC2 isoform carriage
	GC2 carriage	Vitamin D3	Placebo	Vitamin D3	p^b^	Placebo	p	Vitamin D3	p	Placebo	p	Vitamin D3	p	Placebo	p	P for interaction^c^
Total 25(OH)D_3_, ng/mL	0	266	89	29.9		30.4		41.2		28.9		11.3		-1.5		p=0.63
	1	130	51	27.0*		25.6*		37.4*		24.0*		10.5		-1.6		
	2	18	5	25.3		21.8		35.0*		16.2*		9.7		-5.6		
					0.063		0.018		0.002		0.001		0.640		0.062	
Directly Measured Free 25(OH)D_3_, pg/mL	0	266	89	43.4		44.8		58.6		38.7		15.3		-6.1		p=0.12
	1	130	51	43.8		42.2		54.9		38.4		11.1		-3.9		
	2	18	5	40.1		32.2		56.8		28.0		16.7		-4.2		
					0.81		0.47		0.28		0.27		0.32		0.84	
Bikle’s Calculated Free 25(OH)D_3_, pg/mL	0	266	89	9.6		9.5		13.2		9.0		3.7		-0.6		p=0.78
	1	130	51	9.1		8.5		12.7		8.0		3.5		-0.4		
	2	18	5	9.3		8.8		12.8		6.9		3.5		-1.7		
					0.520		0.240		0.370		0.075		0.880		0.096	
24,25(OH)_2_D_3_/25(OH)D_3_	0	266	89	5.9E-02		5.9E-02		7.0E-02		5.5E-02		1.2E-02		-3.5E-03		p=0.81
	1	130	51	5.9E-02		5.8E-02		7.1E-02		5.4E-02		1.2E-02		-4.2E-03		
	2	18	5	5.8E-02		6.0E-02		7.3E-02		5.8E-02		1.4E-02		-2.5E-03		
					0.96		0.97		0.62		0.89		0.64		0.78	

*Indicates statistical significance at p<0.05 when compared with reference level *GC*2 carriage=0.

^a^Values are the adjusted means derived from the multivariable linear model adjusted for *GC*1s isoform carriage and the other previously listed covariates.

^b^P-value from the multivariate Wald test of *GC*2 isoform covariate.

^c^P-value from the test of interaction between *GC*2 isoform and treatment assignment in a multivariable model including all participants (vitamin D_3_ and placebo).

**Table 3:** Changes in total and free serum 25(OH)D3 in response to vitamin D3 supplementation according to GC1s isoform carriage^a^ .

		N		Baseline				16 week				Change after 16 weeks				Treatment effect modification by GC1s isoform carriage
	GC1s carriage	Vitamin D3	Placebo	Vitamin D3	p^b^	Placebo	p	Vitamin D3	p	Placebo	p	Vitamin D3	p	Placebo	p	P for interaction^c^
Total 25(OH)D_3_, ng/mL	0	164	50	28.7		27.5		39.2		27.2		10.5		-0.3		p=0.74
	1	161	72	28.2		29.0		39.9		26.9		11.6		-2.1		
	2	89	23	29.9		29.1		40.3		25.4		10.4		-3.7		
					0.60		0.81		0.78		0.83		0.53		0.22	
Directly Measured Free 25(OH)D_3_, pg/mL	0	164	50	40.8		46.7		55.5		40.4		14.7		-6.3		p=0.41
	1	161	72	45.0		41.0		56.6		36.7		11.5		-4.3		
	2	89	23	44.7		42.4		62.5		37.0		17.8		-5.3		
					0.57		0.7		0.31		0.75		0.35		0.92	
Bikle’s Calculated Free 25(OH)D_3_, pg/mL	0	164	50	9.4		8.7		12.9		8.6		3.5		-0.2		p=0.33
	1	161	72	9.4		9.5		13.2		8.9		3.9		-0.8		
	2	89	23	9.6		9.2		13.0		8.2		3.3		-0.7		
					0.90		0.54		0.68		0.63		0.38		0.28	
24,25(OH)_2_D_3_/25(OH)D_3_	0	164	50	5.9E-02		5.9E-02		7.0E-02		5.5E-02		1.2E-02		-3.5E-03		p=0.85
	1	161	72	5.8E-02		5.8E-02		7.1E-02		5.5E-02		1.2E-02		-2.6E-03		
	2	89	23	5.6E-02		5.6E-02		6.6E-02		4.8E-02		9.6E-03		-8.0E-03		
					0.67		0.5		0.15		0.064		0.45		0.19	

^a^Values are the adjusted means derived from the multivariable linear model adjusted for *GC*2 isoform carriage and the other previously listed covariates.

^b^P-value from the multivariate Wald test of *GC*1s isoform covariate.

^c^P-value from a test of interaction between *GC*1s isoform and treatment assignment in a multivariable model including all participants (vitamin D_3_ and placebo).

**Figure 1:**
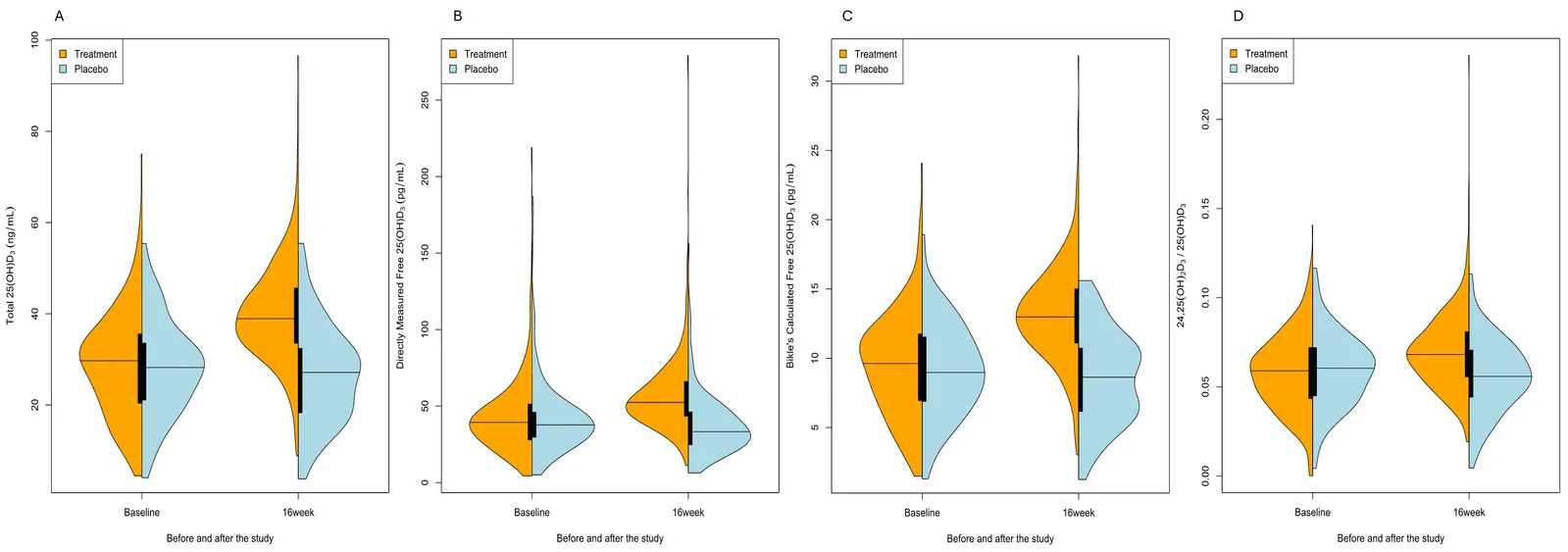
A panel of violin plots showing the distributions of the outcome biomarkers at baseline and 16 weeks according to treatment group. A violin plot is split into two sections representing baseline” and 16 week data. Within each section, there are two violin shapes: one light blue representing the treatment group and one orange representing the placebo group. The width of the violins reflects the data density. Black lines within the violins indicate the median value. **(A)** Total 25(OH)D_3_ (ng/mL), **(B)** Directly Measured Free 25(OH)D_3_ (pg/mL), **(C)** Calculated Free 25(OH)D_3_ (pg/mL), and **(D)** 24,25(OH)_2_D_3_/25(OH)D_3_ (VDMR). Each panel displays the distribution of values for both treatment groups at both time points.

### 3.4 Free 25(OH)D_3_

Directly measured or calculated free 25(OH)D_3_ concentration did not vary according to GC isoform at either baseline or endpoint (16 weeks). Given that the total 25(OH)D_3_ was lower with GC2 isoform carriage at baseline and endpoint, but free 25(OH)D_3_ did not differ, it follows that the free fraction of 25(OH)D_3_ was higher among individuals carrying GC2 isoform (**Supplementary Tables 2**, **3**).

In the vitamin D_3_ group, free 25(OH)D_3_ concentration increased by 13.93 (± 28.68) pg/mL and 3.64 (± 3.14) pg/mL for measured free and calculated free, respectively, whereas it remained stable in the placebo arm (**Figures 1B, C**). As hypothesized, the change in free 25(OH)D_3_ with supplementation was not associated with GC isoform.

### 3.5 24,25(OH)_2_D_3_-to-25(OH)D_3_ ratio (VDMR)

As hypothesized, there were no significant differences in the VDMR according to GC isoform carriage at either baseline or endpoint (**Tables 2**, **3**). Vitamin D_3_ supplementation was associated with a modest increase in the VDMR (**Figure 1D**), but the change did not vary according to isoform.

## 4 Discussion

In this study, we investigated whether GC isoforms modify the effects of 16 weeks of vitamin D_3_ supplementation on circulating concentrations of total and free 25(OH)D_3_. Based on the results, individuals carrying GC2 alleles had lower baseline and 16-week measurements of total serum 25(OH)D_3_, but not free 25(OH)D_3_ concentration. GC1s isoform carriage was not associated with total or free 25(OH)D_3_ concentrations, and neither GC2 nor GC1s carriage was associated with the total or free 25(OH)D_3_ response to vitamin D_3_ supplementation.

Our first finding was that each GC2 allele was associated with lower total 25(OH)D_3_ and a greater free fraction of 25(OH)D_3_. This has been shown previously when free 25(OH)D_3_ concentrations were calculated or measured by immunoassay, and our results confirm the prior reports using direct measurement of free 25(OH)D_3_ by mass spectrometry (37–39).

Our second finding was that neither the total nor free 25(OH)D_3_ response to vitamin D_3_ supplementation varied according to GC isoform. This finding is consistent with the study by Sollid et al., who also evaluated total and free 25(OH)D responses to vitamin D supplementation according to vitamin D binding protein phenotype (37). In that study, no statistically significant differences in either total or free 25(OH)D response were detected between GC isoform groups. However, the authors observed a trend toward a smaller increase in total 25(OH)D among individuals carrying the GC2 isoform and suggested that a larger sample size might be needed to determine whether this represented a true association. Despite evaluating a larger multiethnic cohort, we similarly found no statistically significant differences in either total or free 25(OH)D_3_ response according to GC isoform.

This result differed from our original hypothesis. Based on prior evidence, we expected the change in total 25(OH)D_3_ to differ between isoform groups because GC isoforms are associated with differences in the free fraction of circulating 25(OH)D_3_ and may influence the systemic clearance of the metabolite. In a prior study of 25(OH)D_3_ systemic clearance in individuals with and without chronic kidney disease, we administered deuterated 25(OH)D_3_ intravenously and detected faster clearance among those with GC2 isoform (17). We hypothesized that faster clearance with GC2 carriage would result in an attenuated total 25(OH)D_3_ response to oral vitamin D_3_ in INVITe, which was not supported by the results of this analysis. One possible explanation for this discrepancy is that differences in clearance according to GC isoform are not substantial enough to translate to differences in supplementation response in every study, given that other factors, including other genetic variants, contribute to variability in response. Indeed, we previously identified the common genetic variants that were associated with the total 25(OH)D response to supplementation in INVITe, and none were within or adjacent to *GC* gene (24). That analysis corrected for many multiple comparisons, which has not been the case in prior studies investigating how the common GC isoforms modify the effect of supplementation on the change in 25(OH)D. Because prior studies have not consistently shown a difference in supplementation response by GC isoform and any reported differences have generally been small, our null results support that any isoform-related effects, if present, are likely modest.

While rs4588 and rs7041 variants (main GC isoforms) may have only a modest impact on the circulating half-life of 25(OH)D_3_, they may more substantially impact the efficiency of translocation of vitamin D_3_ from the epidermis to the circulation and therefore modify the 25(OH)D_3_ response to sun exposure. VDBP is required for transporting vitamin D_3_ from the skin to the bloodstream after sun exposure (40). Variation in VDBP concentration and affinity may therefore directly affect the efficiency of vitamin D transfer from the epidermis to the circulation. In a recent analysis of UK biobank data, the association between rs4588 and circulating 25(OH)D concentration was found to be associated with season and magnified with increased time spent outdoors (41).

In this study, we observed poor agreement between free 25(OH)D_3_ determined by LC-MS/MS and calculated values determined using a calculation-based method. Calculation methods for estimating free 25(OH)D concentration assume uniform binding affinity across the population, an assumption that is frequently inaccurate. In addition to variant binding proteins, clinical conditions, medications, and physiological factors can significantly alter binding dynamics, leading to inaccurate estimates and systematic bias (9, 23, 26). These limitations highlight the need for methods for direct measurement of free 25(OH)D.

The strengths of this study include that it uses data from a large, randomized controlled trial that was uniquely designed to evaluate both clinical and genetic modifiers of vitamin D treatment response among a multiethnic population from multiple geographic sites. Excellent participant retention and adherence to supplementation enhanced the reliability and interpretability of the findings. Measurement of total 25-hydroxyvitamin D and vitamin D–binding protein concentrations by gold-standard LC-MS/MS methodology ensured analytical accuracy and precision. Direct quantification of free 25(OH)D_3_ concentration by LC-MS/MS is a novel approach that allowed us to corroborate findings based on calculated free 25(OH)D_3_. Moreover, validation of genotype assignment through concordance between LC-MS/MS–based and microarray-derived data further strengthens the integrity of the analyses.

This study also has some limitations. It was an exploratory analysis that leveraged the INVITe randomized controlled trial design and biospecimen archive; however, the original study was not specifically designed to address this study’s objectives. Thus, the findings should be interpreted with caution and considered preliminary until replicated in future, dedicated trials. Given the randomized controlled trial design, these findings apply to our multi-ethnic study population of older U.S. adults and may not be generalizable to different settings and populations with different ages or health statuses. Additionally, measured free 25(OH)D concentrations in the present study were higher than those reported in some previous studies using immunoassay-based methods and an LC-MS/MS method utilizing a different sample preparation approach (42, 43). The reasons for these differences remain uncertain but may reflect methodological differences among assays. These observations underscore the need for assay harmonization and development of reference measurement procedures for free 25(OH)D measurement.

In summary, although GC isoforms of vitamin D binding protein have shown some of the strongest associations with circulating 25(OH)D concentrations in GWAS studies, our data indicate that they do not significantly associate with the increment of 25(OH)D gained in response to supplementation in this population. This suggests that GC isoforms may play a greater role in determining baseline vitamin D status rather than mediating individual responsiveness to oral supplementation.

Future research should assess whether GC isoforms (or the SNPs that determine them) influence the change in 25(OH)D following UVB exposure, a distinct physiological pathway for vitamin D synthesis and transport. Because circulating 25(OH)D concentration is influenced by numerous genetic loci, future efforts to personalize vitamin D dosing might benefit from the integration of polygenic risk scores. Such approaches could help refine strategies for individualized supplementation and guide personalized vitamin dosing strategies.

## 5 Equations

The equations should be inserted in editable format from the equation editor.


$$free\;vitamin\;D\;metabolite=\frac{Total\;vitamin\;D\;metabolite}{1+[Ka_{alb}*albumin]+[Ka_{DBP}*DBP]}$$

## Data Availability

The original contributions presented in the study are publicly available. These data can be found here: https://mesa-nhlbi.org/.
