# Supplementary material for: Influence of vitamin D binding protein isoforms on the total and free serum 25-hydroxyvitamin D responses to vitamin D supplementation in older adults in the Multi-Ethnic Study of Atherosclerosis
Source: Front Endocrinol (Lausanne). 2026 Oct 29;17:1910020. doi: 10.3389/fendo.2026.1910020 (PMC13755556; doi:10.3389/fendo.2026.1910020)
Supplement: Supplementary file 1 [file SupplementaryFile1.docx]

**Influence of Vitamin D Binding Protein Isoforms on the Total and Free Serum 25-hydroxyvitamin D Responses to Vitamin D Supplementation in Older Adults in the Multi-Ethnic Study of Atherosclerosis**

**Supplemental Figures and Tables**

**Table S1.** Mapping of GC rs7041 and rs4588 Genotypes to Classical Isoforms (GC1F, GC1S, and GC2).

**Figure S1**. Participant Flow in the Multi-Ethnic Study of Atherosclerosis Individualized Response to Vitamin D Treatment Trial.

**Figure S2**. Compared the free 25(OH)D_3_ LC-MS/MS measured method and Bikle’s calculated method using Deming regression analysis.

**Figure S3.** Compared the free 25(OH)D_3_ LC-MS/MS measured method and Bikle’s calculated method using Bland-Altman analysis.

**Table S2**. Percent free 25(OH)D_3_ at baseline and 16 weeks according to GC2 carriage.

**Table S3.** Percent free 25(OH)D_3_ at baseline and 16 weeks according to GC1s carriage.

**Table S1.** Mapping of GC rs7041 and rs4588 Genotypes to Classical Isoforms (GC1F, GC1S, and GC2)**.**

| GC isoform | rs7041 allele | rs4588 allele | Amino acid change relative to GC1f |
| --- | --- | --- | --- |
| GC1f | T (Asp) | C (Thr) | Reference |
| GC1S | G (Glu) | C (Thr) | Asp🡪Glu |
| GC2 | T (Asp) | A (Lys) | Thr🡪Lys |

**Figure S1**. Participant Flow in the Multi-Ethnic Study of Atherosclerosis Individualized Response to Vitamin D Treatment Trial.

**Figure S2**. Compared the free 25(OH)D_3_ LC-MS/MS measured concentrations and Bikle’s calculated concentrations using Deming regression analysis. The blue line represents the fitted Deming regression line (y=11.3x-62.9), which accounts for measurement errors in both variables. The red line indicates the identity line (y=x), serving as a reference for perfect agreement.

**Figure S3**. Compared the free 25(OH)D_3_ LC-MS/MS method and Bikle’s calculation method using Bland-Altman analysis. The Bland-Altman plot is used to assess the agreement between two quantitative methods of measurement. Difference between methods = Measured value – Bikle’s calculated value in pg/ml. The black line indicates the mean of the difference (mean difference=34pg/mL). Dashed red lines represent the limits of agreement (mean difference ±1.96 × standard deviation of the differences), which are the upper and lower 95% limits of agreement.

**Table S2.** Percent free 25(OH)D_3_ at baseline and 16 weeks according to GC2 carriage.

|  |  | **N** | | **Baseline** | | **16 week** | | | |
| --- | --- | --- | --- | --- | --- | --- | --- | --- | --- |
|  | GC2 carriage | Treatment | Placebo | All participants | p | Treatment | p | Placebo | p |
| Measured free 25(OH)D_3_/total 25OHD_3_*100 | 0 | 266 | 89 | 0.152% |  | 0.146% |  | 0.146% |  |
|  | 1 | 130 | 51 | 0.166%* |  | 0.150% |  | 0.162% |  |
|  | 2 | 18 | 5 | 0.156% |  | 0.159% |  | 0.164% |  |
|  |  |  |  |  | 0.008 |  | 0.32 |  | 0.11 |
| Bikle's Calculated Free 25(OH)D_3_/Total 25(OH)D_3_*100 | 0 | 266 | 89 | 0.033% |  | 0.053% |  | 0.031% |  |
|  | 1 | 130 | 51 | 0.034%* |  | 0.053% |  | 0.032% |  |
|  | 2 | 18 | 5 | 0.037%* |  | 0.061% |  | 0.032% |  |
|  |  |  |  |  | 1.10E-13 |  | 0.57 |  | 0.69 |

* indicates statistical significance at p<0.05 when compared with reference level GC2 carriage=0.

P-value from the multivariate Wald test of GC2 isoform covariate.

Both the numerator and the denominator are expressed in ng/mL in the calculation.

**Table S3.** Percent free 25(OH)D_3_ at baseline and 16 weeks according to GC1s carriage.

|  |  | **N** | | **Baseline** | | **16 week** | | | |
| --- | --- | --- | --- | --- | --- | --- | --- | --- | --- |
|  | GC1S carriage | Treatment | Placebo | All participants | p | Treatment | p | Placebo | p |
| Measured free 25(OH)D_3_/total 25OHD_3_*100 | 0 | 164 | 50 | 0.148% |  | 0.142% |  | 0.146% |  |
|  | 1 | 161 | 72 | 0.153% |  | 0.144% |  | 0.162% |  |
|  | 2 | 89 | 23 | 0.155% |  | 0.159% |  | 0.164% |  |
|  |  |  |  |  | 0.71 |  | 0.35 |  | 0.43 |
| Bikle's calculated Free 25(OH)D_3_/Total 25(OH)D_3_*100 | 0 | 164 | 50 | 0.033% |  | 0.052% |  | 0.033% |  |
|  | 1 | 161 | 72 | 0.033% |  | 0.054% |  | 0.031% |  |
|  | 2 | 89 | 23 | 0.033% |  | 0.049% |  | 0.028% |  |
|  |  |  |  |  | 0.2 |  | 0.53 |  | 0.16 |

* indicates statistical significance at p<0.05 when compared with reference level GC1s carriage=0.

P-value from the multivariate Wald test of GC1s isoform covariate.

Both the numerator and the denominator are expressed in ng/mL in the calculation.
